# Poster Session I - A119 ONE VERSUS TWO FECAL IMMUNOCHEMICAL TESTS IN THE DETECTION OF ADVANCED COLORECTAL NEOPLASIA

**DOI:** 10.1093/jcag/gwaf042.119

**Published:** 2026-02-13

**Authors:** B McGrath, S Antle, G Doyle, M Borgaonkar, J McGrath

**Affiliations:** Memorial University of Newfoundland, St. John’s, Canada; Memorial University of Newfoundland, St. John’s, Canada; Memorial University of Newfoundland, St. John’s, Canada; Memorial University of Newfoundland, St. John’s, Canada; Memorial University of Newfoundland, St. John’s, Canada

## Abstract

**Background:**

FIT is widely used for CRC screenings. Most Canadian programs only use one FIT sample when determining the need for a colonoscopy. It is unclear if an analysis of both samples in a standard two-sample kit improves detection of CRC and advanced adenomas.

**Aims:**

To determine the positive predictive value (PPV) of one versus two abnormal FIT in the detection of colorectal cancer and advanced adenomas.

**Methods:**

Over a five year period from 2020 to 2025, 84,489 completed FIT kits were received by the Newfoundland and Labrador colon cancer screening program. A participant completed two FIT samples and if one FIT was positive ( > =100ng/ml) a colonoscopy was arranged. Data collected included demographics, colonoscopy, pathology, and quantitative FIT results. PPV of one versus two abnormal FITs was calculated.

**Results:**

10,237 patients (12.1%) were positive when the FIT was analyzed. 9,203 colonoscopies were completed and 249 (2.7%) colorectal cancers were detected. The number of advanced adenomas was 26.2% and overall Adenoma Detection Rate was **61.1%.**

Of the 249 cancers detected, 183 patients had both FIT samples positive while 66 patients had one FIT sample positive and one negative, 33 of 249 (13.3%) of all cancers would have been missed if only one FIT was analyzed. For 2415 participants with advanced adenomas 1194 had one FIT positive and 1219 had both positive. If only one FIT test was analyzed 597 advanced adenomas would have been missed.

The positive predictive value (PPV) of one vs. two positive FITs for cancer was 2.4% vs 2.7% (p value < 0.01) and the PPV for advanced adenoma was 19.8% vs. 26.2% (*p* value < 0.01). When the first FIT was negative, the second positive FIT detected 33 additional cancers and an extra 592 advanced adenomas. Absolute FIT values were also higher for patients with two FIT positive compared to one FIT positive for both cancer (6449 vs. 5337) and advanced adenomas (2243 vs. 878).

**Conclusions:**

PPV of two positive FITs is superior to one positive FIT for both colorectal cancer and advanced adenomas. There was an added value when participants completed a second FIT kit.

**Funding Agencies:**

None

